# Immunohistochemical Evaluation of RUNX2, Osteocalcin, and Vimentin in Feline Primary Pulmonary Carcinomas

**DOI:** 10.3390/vetsci13090980

**Published:** 2026-09-17

**Authors:** Alexandra Morar, Corina Toma, Dragoș Hodor, Mara-Georgiana Haralambie, Iulia Pană, Alexia-Teodora Hoța, Diana Bochynska, Claudiu Gal, Cornel Cătoi

**Affiliations:** 1 Department of Veterinary Pathology, Faculty of Veterinary Medicine, University of Agricultural Sciences and Veterinary Medicine Cluj-Napoca, 400372 Cluj-Napoca, Romania; alexandra.morar@usamvcluj.ro (A.M.); dragos.hodor@usamvcluj.ro (D.H.); mara-georgiana.haralambie@usamvcluj.ro (M.-G.H.); iulia.pana@student.usamvcluj.ro (I.P.); alexia-teodora.hota@usamvcluj.ro (A.-T.H.); claudiu.gal@synevovet.ro (C.G.); cornel.catoi@usamvcluj.ro (C.C.); 2 Department of Veterinary Pathology, Ross University School of Veterinary Medicine, Basseterre 00265, Saint Kitts and Nevis; dbochynska@rossvet.edu.kn; 3 Department of Veterinary Pathology, Synevovet, Industriilor 25, 077040 Chiajna, Romania

**Keywords:** feline primary pulmonary carcinoma, feline lung-digit syndrome, digital metastasis, RUNX2, osteocalcin, immunohistochemistry, adenosquamous carcinoma

## Abstract

Feline primary pulmonary carcinomas (PPCs) occasionally exhibit a distinctive metastatic tropism for the distal digits, clinically recognized as feline lung-digit syndrome or MODAL syndrome. The molecular mechanisms driving this unique behavior remain poorly understood. This retrospective study evaluated 18 feline PPCs to investigate the expression of bone-related markers (RUNX2, osteocalcin), vimentin, Ki-67, MCK, and TTF-1. Cases were classified into PPCs with (*n* = 5) and without (*n* = 13) documented digital metastases. Adenosquamous carcinoma was the predominant histological subtype (61.1%); all tumors expressed MCK and TTF-1. While osteocalcin and aberrant vimentin expression were detected across both groups, they did not differ significantly between groups. Conversely, RUNX2 nuclear immunoreactivity was significantly elevated in cases with digital metastasis (*p* = 0.006). Furthermore, the evaluation of matched primary and metastatic lesions demonstrated that digital metastases retained the histological and immunophenotypic profiles of their primary tumors. Overall, this first characterization demonstrates that higher RUNX2 immunoreactivity was observed in primary pulmonary carcinomas from cats with documented digital metastasis. However, without the evaluation of extra-digital metastases, it remains undetermined whether this reflects a specific tropism for the digits or a general metastatic competence.

## Simple Summary

Primary lung cancer in cats is rare but often spreads directly to the digits, a condition known as feline lung-digit syndrome. The biological reasons for this unique bone-targeted spread remain poorly understood. In this study, we examined lung tumors from 18 cats, including 5 cases where the cancer had spread to the digits. We tested these samples for RUNX2, osteocalcin, and vimentin. We found that tumors spreading to the toes had significantly higher levels of RUNX2 protein compared to those that did not. Ultimately, this study provides the first descriptive data showing that higher RUNX2 immunoreactivity is observed in primary pulmonary carcinomas from cats with documented digital metastasis.

## 1. Introduction

Primary pulmonary tumors are uncommon in cats but are clinically significant due to their aggressive biological behavior, high metastatic potential, and generally unfavorable prognosis [1,2,3]. These epithelial neoplasms, predominantly comprising adenocarcinomas and adenosquamous carcinomas, typically affect older felines [1,2,3,4]. The etiopathogenesis remains largely unknown, and clinical recognition is frequently delayed until the disease reaches an advanced stage or metastatic lesions become apparent [1,3,5,6]. A distinctive metastatic pattern in this species is the feline lung-digit syndrome, which is now recognized within the broader feline MODAL (muscular, ocular, digital, aortic, and lung) syndrome [6,7]. This syndrome is characterized by a unique metastatic tropism for the distal phalanges [6,7].

Although several molecular and immunohistochemical features of feline primary pulmonary carcinomas (PPCs) have been documented, including TP53 and HER2 alterations, a lack of human-like K-RAS mutations, and the diagnostic utility of TTF-1, MCK, and Ki-67 [8,9,10,11,12,13], the biological mechanisms driving this digital tropism remain poorly understood. One potential mechanism facilitating such dissemination is epithelial–mesenchymal transition (EMT), a dynamic biological process wherein epithelial tumor cells acquire mesenchymal characteristics and increased invasive properties [14]. While a comprehensive evaluation of EMT requires the assessment of specific transcription factor networks, an initial and partial characterization frequently involves evaluating the aberrant expression of vimentin, a mesenchymal intermediate filament [15,16]. While vimentin expression has been reported in feline PPCs, its specific relationship with digital metastasis remains incompletely characterized [14,15,16].

Furthermore, successful metastatic colonization requires adaptation to the target tissue microenvironment, conceptually described by the seed and soil hypothesis [17,18,19]. The preferential involvement of the distal phalanges in feline MODAL syndrome suggests that pulmonary carcinoma cells may express bone-associated proteins, potentially reflecting a phenotypic adaptation associated with survival and proliferation within the osseous environment [20]. Runt-related transcription factor 2 (RUNX2), a central transcription factor in osteoblast differentiation, is associated with tumor progression, invasion, and metastatic behavior in various human epithelial cancers [20,21,22,23]. Similarly, increased expression of osteocalcin, a downstream component of osteogenic programs, has been investigated in relation to bone-associated metastatic behavior [17,18,19]. The role of these osteogenic molecular programs in feline PPCs, however, remains unexplored.

Therefore, the objective of the present study was to characterize and compare the histological and immunohistochemical features of feline PPCs with and without digital metastases. Specifically, the study investigated vimentin expression as a preliminary marker of an EMT-associated phenotype, alongside RUNX2 and osteocalcin as markers of osteogenic cellular programs. Proliferative activity was estimated via Ki-67 immunolabeling. Finally, paired primary pulmonary tumors and their corresponding digital metastatic lesions were evaluated to determine whether these immunophenotypic characteristics are maintained during metastatic progression. We hypothesized that feline PPCs with digital metastases would exhibit significantly higher expression of RUNX2 and osteocalcin compared to non-metastatic tumors, reflecting a potential molecular association with digital tissue colonization. Furthermore, we hypothesized that increased vimentin expression would be more prevalent in the metastatic group, suggesting a potential role for cellular plasticity in disease progression.

## 2. Materials and Methods

### 2.1. Case Selection

A total of eighteen (*n* = 18) feline PPCs diagnosed between 2020 and 2025 were retrospectively retrieved from the archives of the Department of Veterinary Pathology, University of Agricultural Sciences and Veterinary Medicine Cluj-Napoca, Romania, and the SynevoVet Laboratory, Bucharest, Romania. The study included all eligible PPCs, including five (*n* = 5) cases with associated digital metastases, that were part of MODAL syndrome. Tissue samples from sixteen cases were obtained during routine postmortem examination and two were excisional biopsy specimens (lobectomy). When available, corresponding digital metastases were also collected for histopathological and immunohistochemical evaluation. Metastatic lesions at other sites were also recorded.

Patient signalment (breed, age, and sex) together with gross and histopathological findings were retrieved from the medical records.

Eligibility criteria were: (1) availability of formalin-fixed, paraffin-embedded (FFPE) tissue containing sufficient neoplastic tissue for histological and immunohistochemical analyses; (2) adequate tissue preservation, with well-preserved morphology and minimal or no autolysis; (3) a confirmed diagnosis of primary pulmonary carcinoma based on histological features and immunohistochemical expression of MCK and TTF-1, with no evidence of an extrapulmonary primary tumor, based on clinical records, diagnostic imaging (when available), and/or complete necropsy; and (4) availability of patient signalment.

Cases were excluded if they represented metastatic pulmonary carcinomas, primary pulmonary non-epithelial neoplasms, tumors with an uncertain diagnosis following histological and immunohistochemical evaluation or had incomplete clinicopathological data.

### 2.2. Histopathological Examination

All formalin-fixed paraffin-embedded (FFPE) tissue specimens were sectioned at 2–3 μm thickness. For digital metastatic specimens containing bone, tissue decalcification was performed prior to paraffin embedding using a standard EDTA-based solution. For routine histological evaluation, sections were stained with hematoxylin and eosin (H&E). Histological examination of all samples was performed using an Olympus BX41 bright-field microscope (Olympus, Tokyo, Japan), and representative histological fields were captured using an Olympus UC30 (Olympus, Tokyo, Japan) digital camera and Stream Basic imaging software, version 1.5.1. In addition, stained slides were digitally scanned to enable whole-slide visualization and digital image analysis. QuPath version 0.7.0 and ImageJ software version 1.54p were used for the selection and evaluation of representative regions of interest and for image-based analyses, where applicable.

Histological classification of the included pulmonary neoplasms was performed as previously described [3]. Additional morphological parameters were evaluated semiquantitatively, including mitotic count, vascular invasion, desmoplastic stroma, intratumoral necrosis, inflammatory infiltrates, anisocytosis, and anisokaryosis. Mitotic figures were manually counted within a standardized area of 2.37 mm^2^. This area corresponds to 10 high-power fields (HPFs) evaluated at 400× magnification using an Olympus BX41 microscope (Olympus, Tokyo, Japan), equipped with a 40× objective and 10× eyepieces with a field number (FN) of 22. Vascular invasion was graded according to the number of affected vessels (few: <5 vessels; moderate: 5–10 vessels; marked: >10 vessels). Desmoplastic stroma and intratumoral necrosis were scored based on their extent within the neoplastic area (absent: <1%; mild: 1–10%; moderate: 11–50%; marked: >50%). Inflammatory infiltrates were graded according to the number of inflammatory cells within the tumor area (absent: <1 cell; mild: 1–15 cells; moderate: 16–50 cells; marked: >50 cells). Anisocytosis and anisokaryosis were assessed according to the proportion of affected neoplastic cells (absent: <1%; mild: 1–15%; moderate: 16–49%; marked: >50%) [3].

### 2.3. Study Groups

To investigate clinicopathological and immunophenotypic differences associated with digital metastasis in feline MODAL syndrome, the 18 primary pulmonary carcinomas (PPCs) were classified into two groups according to the presence or absence of histologically confirmed digital metastasis. The PPC-DM group (PPCs with digital metastasis; *n* = 5) comprised all cases with histologically confirmed metastasis to the distal digit. The PPC-NDM group (PPCs without documented metastasis; *n* = 13) comprised cases in which no metastatic lesions were identified based on the available clinical records and/or postmortem examination. As this study followed a retrospective design, advanced cross-sectional imaging and standardized comprehensive staging were not uniformly available for all feline patients. Consequently, the non-metastatic designation was based strictly on the absence of clinically or pathologically documented lesions, recognizing that the potential presence of early, occult microscopic dissemination cannot be completely excluded.

Additional metastatic lesions at other anatomical sites were identified in three cats included in the PPC-DM group. These included intestinal, regional lymph node, and subcutis. While the initial scope of this investigation focused specifically on the primary tumor-digital metastasis axis, archival tissue for the documented extra-digital sites was ultimately exhausted and unavailable for immunohistochemical evaluation.

For cases in the PPC-DM group, only the digital metastatic lesions were evaluated together with their corresponding primary pulmonary tumors by immunohistochemistry. Paired comparisons were performed to assess the expression of the investigated immunomarkers between the primary pulmonary tumors and their corresponding digital metastatic lesions.

### 2.4. Immunohistochemistry

Immunohistochemical analysis was performed on FFPE tissue sections of feline PPCs and digital metastases, using a panel of antibodies for the detection of MCK, TTF-1, vimentin, osteocalcin, RUNX2, and Ki-67 (Table 1).

Immunostaining for MCK, TTF-1, vimentin, and Ki-67 was performed using an automated Leica Bond Max system (Leica Biosystems, Melbourne, Australia) according to the manufacturer’s recommended protocols.

Immunohistochemical staining for osteocalcin and RUNX2 was performed manually. For manual staining, heat-induced epitope retrieval was performed using ethylenediaminetetraacetic acid (EDTA) buffer for RUNX2, whereas enzymatic antigen retrieval was used for OC. Following blocking of endogenous peroxidase activity with hydrogen peroxide and a protein-blocking step, sections were incubated with the respective primary antibodies under optimized conditions. For all antibodies, immunoreactivity was visualized using 3,3′-diaminobenzidine chromogen, followed by counterstaining with Mayer’s hematoxylin.

Appropriate positive tissue controls were included for each marker (Table 1). Negative controls were obtained by replacing the primary antibody with non-immune serum from the corresponding species.

All immunohistochemical evaluations were performed blindly by two independent observers. Discrepancies in the initial independent scoring were subsequently resolved through consensus review using a multi-head microscope.

To minimize technical variability, immunohistochemical staining batches were balanced to include cases from both the PPC-DM and PPC-NDM groups simultaneously. MCK and TTF-1 immunoreactivity were qualitatively assessed as positive or negative and used to confirm epithelial differentiation and pulmonary origin, respectively [3,10,11].

Vimentin expression was evaluated using a semiquantitative scoring system based on the percentage of immunopositive neoplastic cells (0: no immunolabeling; 1: <10%; 2: 10–25%; 3: 26–50%; 4: >50%) [16].

Osteocalcin expression was assessed overall throughout the entire viable tumor area based on the intensity and distribution of cytoplasmic immunolabeling (0: absent; 1: weak and focal; 2: moderate and multifocal; 3: strong and extensive) [17]. For the semiquantitative evaluation of RUNX2, tissue sections were initially examined at low magnification (10×) to identify tumor ‘hot spots’ (representative regions displaying the highest density and proportion of immunopositive neoplastic nuclei). In adenosquamous carcinomas, both the glandular and squamous components were evaluated collectively to generate the final overall immunohistochemical score.

RUNX2 expression was evaluated based on nuclear immunolabeling of neoplastic cells. Staining intensity was scored from 0 to 3 (0: negative; 1: weak; 2: moderate; 3: strong), and the percentage of positive neoplastic cells was scored from 1 to 4 (<25%: score 1; 25–50%: score 2; 50–75%: score 3; >75%: score 4). A staining index (SI) ranging from 0 to 12 was calculated by multiplying the staining intensity score by the percentage score of positive neoplastic cells. Higher SI values reflected greater overall RUNX2 expression and were used as continuous variables for statistical analysis [19]. For the evaluation of Ki-67, slides were first scanned at low magnification to identify tumor ‘hot spots’ (areas of highest proliferative activity). Ki-67 expression was then assessed by manually counting Ki-67-positive neoplastic nuclei and the total number of neoplastic cell nuclei across five high-power fields (the identified hot spot and four adjacent fields) using ImageJ software [12,13]. The proliferative index was calculated as the mean percentage of Ki-67-positive neoplastic cells [12,13].

### 2.5. In Silico Antibody Validation

To validate the selected primary antibodies for feline tissues, in silico sequence homology analyses were performed using UniProt [24], NCBI BLASTp (NCBI BLAST+ v2.15.0) [25], and Clustal Omega (EMBL-EBI, version 1.2.4) [26]. For osteocalcin, the feline sequence (UniProt: P02821) was compared to the bovine immunogen (UniProt: P02820), demonstrating high conservation (87.76% identity, 100% query coverage, E-value: 8 × 10^−32^), with limited, predominantly conservative substitutions. For RUNX2, feline (UniProt: A0ABI7Z5E6) and human (UniProt: Q13950) sequences were aligned. Analysis of the reported RUNX2 immunogen region (amino acids 294–363) yielded 90.0% identity, 71% query coverage, and an E-value of 7 × 10^−31^, exhibiting strong conservation, particularly at the N-terminus. Collectively, these robust homology metrics support the predicted cross-reactivity and biological plausibility of using the anti-bovine osteocalcin and anti-human RUNX2 antibodies in feline tissues. However, formal experimental validation (e.g., Western blot) demonstrating absolute antibody specificity in feline FFPE tissue has not been performed.

### 2.6. Statistical Analysis

Statistical analysis was performed using IBM SPSS Statistics version 32.0.0 (IBM Corp., Armonk, NY, USA). Descriptive statistics were calculated for all investigated variables, including the mean, standard deviation (SD), median, interquartile range (IQR), minimum, and maximum values.

Due to the relatively small sample size and the ordinal nature of the immunohistochemical scoring system, non-parametric statistical tests were applied throughout the study. Differences in vimentin, osteocalcin, RUNX2, and Ki-67 expression between the PPC-NDM and PPC-DM groups were assessed using the Mann–Whitney U test. Rank-biserial correlations (rrb) and their bootstrapped 95% CIs were reported as measures of effect size. Histopathological grading parameters, including vascular invasion, necrosis, anisocytosis/anisokaryosis, and inflammatory infiltrate, were also compared between groups using the Mann–Whitney U test [27,28].

Given the limited sample size (*n* = 5) of the paired primary pulmonary tumors and their corresponding digital metastatic lesions, matched comparisons were evaluated descriptively using individual paired trajectories rather than inferential significance testing. Associations between immunohistochemical markers (vimentin, osteocalcin, RUNX2, and Ki-67), mitotic count, and histopathological grading variables were evaluated using Spearman’s rank correlation coefficient (ρ) for the entire study group. Correlation analyses restricted to the metastatic subgroup were not performed because of the limited sample size. Statistical significance was considered at *p* < 0.05.

During the preparation of this manuscript, artificial intelligence (ChatGPT version GPT-5.6 Luna, OpenAI) was utilized to assist with the generation of graphics and the interpretation of analytical data. All AI-assisted interpretations and visual outputs were critically evaluated, verified, and edited by the authors. The authors assume full responsibility for the final content, accuracy, and integrity of the presented findings.

## 3. Results

### 3.1. Signalment of the Included Cases

Of the eighteen (*n* = 18) cats included in the study, 16 (88.9%) were European Shorthair cats, while the remaining two were a Birman and a Russian Blue (1/18 each; 5.6%). Twelve cats were male (12/18; 66.7%), including eight neutered males (8/12; 66.7%) and four intact males (4/12; 33.3%). Six cats were female (6/18; 33.3%), including five spayed females (5/6; 83.3%) and one intact female (1/6; 16.7%). The median age at diagnosis was 11.0 years (mean, 10.4 years; range, 2–18 years).

### 3.2. Gross Findings

Grossly (Figure S1), all PPCs presented as solitary and unilateral lesions with anatomical distributions detailed in Appendix A, Table A1. The left caudal lung lobe was the most frequently affected site (6/18; 33.3%, followed by the right caudal, right cranial, and left cranial lung lobes (3/18 each; 16.7%). The right middle lung lobe was involved in two cases (2/18; 11.1%), and the accessory lung lobe was affected in one case (1/18; 5.6%).

More than half of the neoplasms (10/18; 55.6%) presented as well-demarcated, discrete nodules, whereas eight cases (8/18; 44.4%) showed an infiltrative growth pattern. On cut section, the masses were predominantly white-to-tan. Most tumors were firm in consistency, although pale, friable areas consistent with necrosis were commonly noted.

In every case of digital metastasis (*n* = 5), only a single digit was affected, with the lesion localized in the distal phalanx. Four cases involved the pelvic limbs, whereas one involved a thoracic limb. Grossly, the lesions presented as solitary nodules causing distension of the affected digit. Nail-bed ulceration with crust formation was also noted in one case. On cut section, the neoplastic tissue was consistently firm, unencapsulated, and white to tan. In all five cases, the metastatic tumors were poorly demarcated and infiltrated the phalangeal bone, resulting in partial effacement of the affected bone.

Additionally, among the cases with digital involvement, extra-digital metastases were systematically documented in three cases. These included metastases to the intestine (*n* = 1), tracheobronchial lymph nodes (*n* = 1), and subcutaneous tissue (*n* = 1). The histological features of these lesions were overall similar to the corresponding PPCs; however, due to the lack of residual tissue in the archival blocks, these specific extra-digital sites were unavailable for further immunohistochemical evaluation.

### 3.3. Histopathological Evaluation

Histological examination of the 18 feline PPCs, including cases with digital metastases (PPC-DM group) and without digital metastases (PPC-NDM), identified two main histological types: adenosquamous carcinoma (ADS) (11/18; 61.1%) and adenocarcinoma (ADC) (7/18; 38.9%) (Figure S2). Within the PPC-NDM (*n* = 13), ADS was diagnosed in seven cases (7/13; 53.8%), whereas six cases (6/13; 46.2%) were classified as ADCs, including four papillary and two acinar subtype. Within the PPC-DM (*n* = 5), four cases (4/5; 80.0%) were classified as ADSs, while one case (1/5; 20.0%) was diagnosed as papillary ADC. Although the sample size limits formal statistical testing, the predominance of ADSs in the metastatic group (80.0%) compared to the non-metastatic group (53.8%) represents a notable descriptive trend toward squamous differentiation in tumors associated with digital colonization.

The ADCs and the glandular components of ADSs exhibited classic morphological features, characterized by cuboidal to columnar cells arranged in papillary projections with delicate fibrovascular stalks or acinar structures containing occasional intraluminal mucin. The squamous components demonstrated variable degrees of keratinization and intercellular bridges.

Mitotic figures were identified in all examined PPCs, with comparable activity between the two groups. PPC-NDM tumors exhibited a mean mitotic count of 4.92 ± 1.44 mitoses/2.37 mm^2^, whereas PPC-DM-associated tumors showed a mean of 5.20 ± 1.10 mitoses/2.37 mm^2^, with considerable overlap between groups. Additional morphological parameters, including desmoplastic stromal response, anisocytosis/anisokaryosis, intratumoral necrosis, and tumor-associated inflammatory infiltrates, were variable but showed no distinct differences between metastatic and non-metastatic tumors (Table A1). Vascular invasion was frequently observed, with involvement of few vessels in 16 cases (88.9%) and moderate involvement in 2 cases (11.1%).

The digital metastatic lesions retained the histological subtype and predominant architectural pattern of their corresponding primary pulmonary tumors, maintaining overall morphological consistency between sites. Additionally, all digital metastases exhibited marked local invasiveness, characterized by infiltration of the dermis, subcutaneous tissues, and nail bed, with progressive replacement of the distal phalanx. Intratumoral necrosis was mild to moderate. Mitotic activity ranged from 3 to 8 mitoses per 2.37 mm^2^ (median, 5), and inflammatory infiltrates were generally mild (4/5; 80%), except for one case showing marked neutrophilic inflammation secondary to ulceration.

### 3.4. Immunohistochemical Evaluation

Immunohistochemically, all primary pulmonary tumors exhibited diffuse, strong cytoplasmic immunolabeling for MCK, confirming their epithelial origin. Strong nuclear TTF-1 immunoreactivity was observed in all cases, supporting the diagnosis of primary pulmonary carcinomas. The complete immunohistochemical profiling for all evaluated cases is summarized in Appendix A, Table A2.

**Figure 1:**
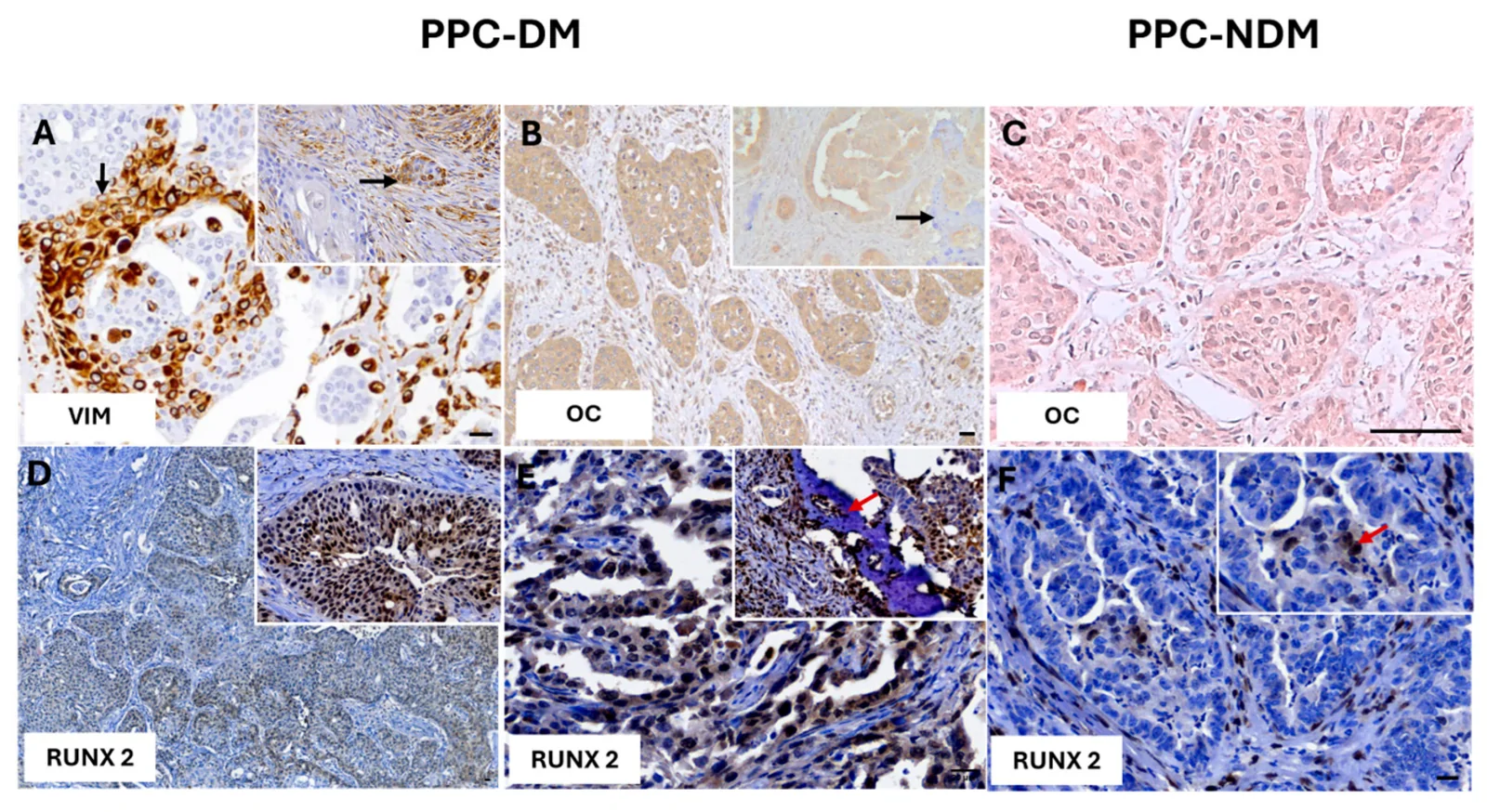
Immunohistochemical characterization of RUNX2, Osteocalcin (OC), and Vimentin (VIM) in feline primary pulmonary carcinomas (PPCs) and matched digital metastases. (**A**,**D**) PPC with digital metastasis (PPC-DM), ADS: (**A**) ADS exhibiting aberrant cytoplasmic VIM immunoreactivity within neoplastic epithelial cells (black arrow). Inset: The matched digital metastasis retains VIM expression (black arrow), scale bar = 20 µm. (**D**) ADS showing diffuse, strong nuclear RUNX2 expression. Inset: The matched digital metastasis retains identical intense nuclear RUNX2 labeling, scale bar = 20 µm. (**B**,**E**) PPC with digital metastasis (PPC-DM), papillary ADC: (**B**) ADS showing diffuse, strong cytoplasmic OC expression. Inset: The corresponding digital metastasis maintains comparable cytoplasmic OC expression (bone-black arrow), scale bar = 20 µm. (**E**) Papillary ADC with strong nuclear RUNX2 expression. Inset: The matched digital metastasis retains intense nuclear RUNX2 immunoreactivity (positive neoplastic cells adjacent to osseous matrix, red arrow), scale bar = 20 µm. (**C**,**F**) PPC without documented metastasis (PPC-NDM): (**C**) ADC exhibiting diffuse cytoplasmic OC expression, scale bar = 50 µm. (**F**) ADC exhibiting weak nuclear RUNX2 immunoreactivity. Inset: Positive nuclei (red arrow), scale bar = 20 µm.

Vimentin immunoreactivity was detected within the cytoplasm of a variable proportion of neoplastic epithelial cells in all evaluated cases, indicating aberrant vimentin expression by the neoplastic epithelial cells. Immunolabeling was heterogeneous and was predominantly observed in small groups of neoplastic cells located, with focal to multifocal distribution throughout the neoplastic tissue. Strong cytoplasmic vimentin immunoreactivity was also consistently observed in stromal mesenchymal cells. Among the primary pulmonary carcinomas, vimentin expression was heterogeneous, with score 1 observed in seven cases (38.9%), score 2 in four cases (22.2%), score 3 in five cases (27.8%), and score 4 in two cases (11.1%). Although vimentin expression was detected in the neoplastic cells of both the PPC-NDM and PPC-DM groups, the PPC-DM (Figure 1A) showed a slightly higher mean vimentin score (2.40 ± 1.14) than the PPC-NDM (2.15 ± 1.07).

Osteocalcin immunoreactivity was present in the majority of tumors, with strong expression (score 3) representing the predominant pattern, observed in 10 of 18 cases (55.6%) (Figure 1B,C). Moderate expression (score 2) was identified in four cases (22.2%), weak expression (score 1) in two cases (11.1%), and two tumors (11.1%) lacked osteocalcin immunoreactivity. The mean osteocalcin score was comparable between the PPC-NDM (2.23 ± 1.01) and the PPC-DM (2.20 ± 1.30). In the adjacent non-neoplastic pulmonary parenchyma, osteocalcin immunoreactivity was also observed in few stromal cells, whereas the remaining pulmonary structures were negative.

For RUNX2 nuclear expression, SI = 2 predominated, occurring in 8 of 18 tumors (44.4%), followed by SI 4 in four cases (22.2%), SI 8 in three cases (16.7%), SI 6 in one case (5.6%), SI 9 in one case (5.6%), and SI 12 in one case (5.6%). PPC-DM showed intense and widespread nuclear immunolabeling (Figure 1D,E), with a mean SI of 7.40 ± 1.95, whereas the PPC-NDM exhibited mild to moderate RUNX2 expression, with a mean staining index (SI) of 3.38 ± 1.89 (Figure 1F). RUNX2 immunolabeling was largely restricted to the neoplastic component, whereas the adjacent non-neoplastic pulmonary parenchyma showed positive nuclei in basal bronchial and bronchiolar epithelium, and occasionally stromal cells.

Ki-67 immunoreactivity was observed as nuclear labeling in neoplastic cells across all evaluated tumors, indicating a high proliferative activity. The mean Ki-67 proliferation index was 65.59% ± 16.07% in the PPC-NDM and 75.78% ± 5.93% in the PPC-DM group.

The digital metastatic lesions retained the core immunophenotypic profile of their corresponding PPC, exhibiting diffuse MCK and TTF-1 immunoreactivity. Vimentin expression remained detectable in all metastatic lesions, with scores ranging from 1 to 4, and osteocalcin expression varied from score 2 to 3. Notably, while primary tumors in the PPC-DM group already exhibited elevated RUNX2 expression, the staining indices increased even further in all matched metastatic lesions, reaching values between 9 and 12.

### 3.5. Statistical Analyses

**Figure 2:**
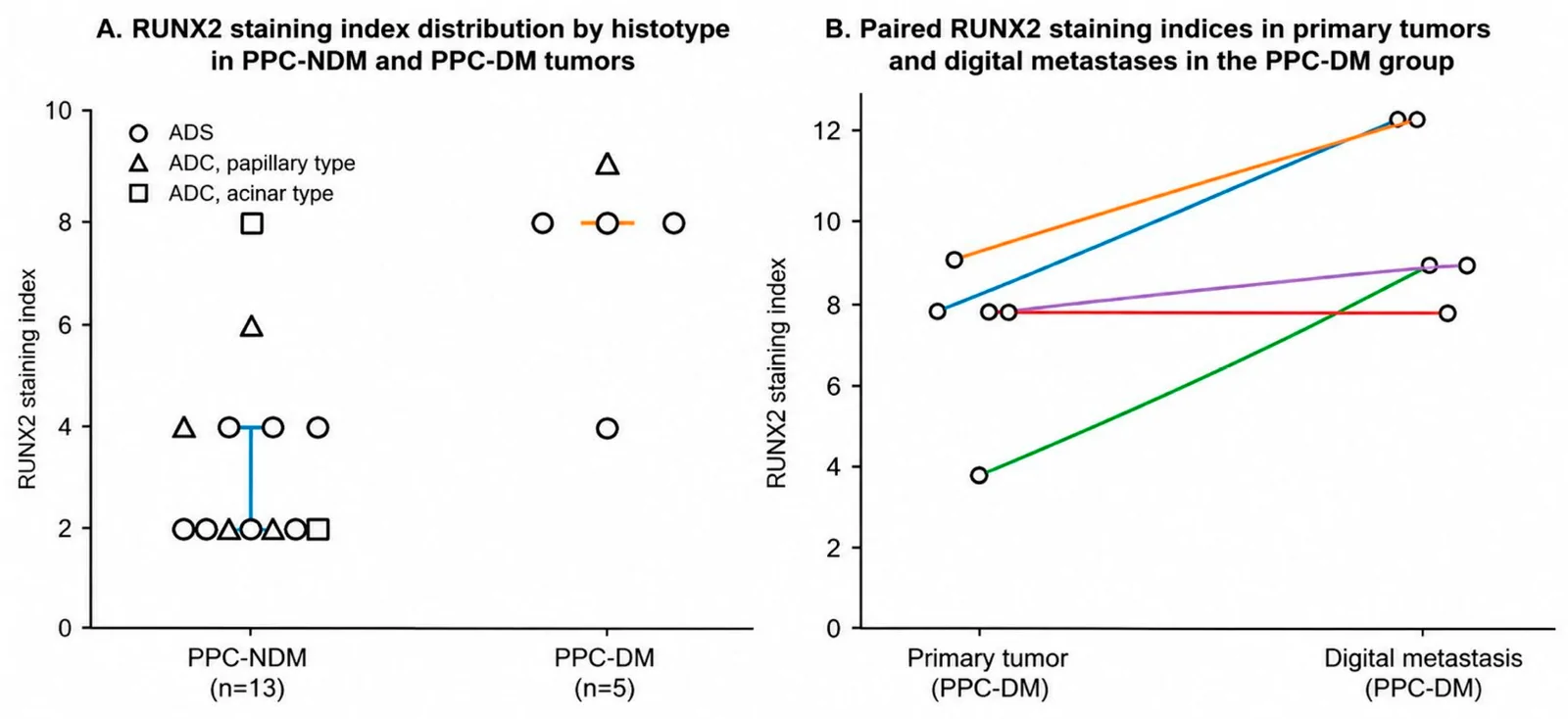
RUNX2 expression in feline primary pulmonary carcinomas and matched digital metastases. (**A**) RUNX2 staining index in primary pulmonary carcinomas without (PPC-NDM; *n* = 13) and with documented digital metastasis (PPC-DM; *n* = 5), with individual values shown by histotype. Bars indicate median and interquartile range. (**B**) Paired RUNX2 staining indices in primary tumors and corresponding digital metastases from PPC-DM cases (*n* = 5).

RUNX2 staining indices were higher in the PPC-DM group than in the PPC-NDM group (U = 5.5, *p* = 0.006), with a large between-group effect (rank-biserial correlation, rrb = 0.831, 95% CI: 0.508–1.000). Ki-67 proliferation indices were also higher in PPC-DM cases, with a moderate-to-large effect estimate, although with substantial uncertainty (U = 12.5, *p* = 0.054; rrb = 0.615, 95% CI: 0.077–1.000). In contrast, between-group effects were small and imprecisely estimated for vimentin (U = 28.5, *p* = 0.719; rrb = 0.123, 95% CI: −0.462–0.662) and osteocalcin (U = 31.5, *p* = 0.957; rrb = 0.031, 95% CI: −0.539–0.554). Given the limited number of matched primary pulmonary tumors and corresponding digital metastatic lesions (*n* = 5 pairs), primary-to-metastasis changes in RUNX2 staining indices were evaluated descriptively and were not interpreted as evidence of equivalent expression or phenotypic stability. Individual RUNX2 observations according to metastatic status and histotype, together with the five matched primary tumor–digital metastasis trajectories, are presented in Figure 2.

Histopathological grading parameters, including vascular invasion, necrosis, anisocytosis/anisokaryosis, and inflammatory infiltrate, showed no statistically significant differences between PPC-NDM and PPC-DM tumors (all *p* > 0.05).

Exploratory Spearman correlation analyses performed across the entire group identified no statistically significant associations between immunohistochemical marker expression and the evaluated histopathological parameters. RUNX2 showed correlations with vascular invasion (ρ = −0.269, *p* = 0.280), necrosis (ρ = −0.162, *p* = 0.520), inflammatory infiltrate (ρ = −0.382, *p* = 0.118), and anisocytosis/anisokaryosis (ρ = 0.125, *p* = 0.621). Osteocalcin was inversely correlated with anisocytosis/anisokaryosis (ρ = −0.370, *p* = 0.130), whereas mitotic count was positively correlated with vascular invasion (ρ = 0.385, *p* = 0.115). These associations were considered exploratory and interpreted cautiously given the small sample size and lack of statistical significance.

## 4. Discussion

The present study characterized the histopathological features and immunohistochemical expression of feline PPCs using a targeted panel of markers (TTF-1, MCK, Vim, OC, RUNX2, and Ki-67). ADS was the most frequent histological subtype and was particularly prevalent among the metastatic tumors [3], the limited sample size precludes determining whether this specific histotype independently drives metastatic behavior.

Aberrant vimentin immunoreactivity was observed across both groups, confirming that the expression of this mesenchymal intermediate filament is a common feature in feline pulmonary carcinoma [3,16]. Because the overall vimentin expression scores were comparable between groups, it does not serve as a sole discriminator for digital metastatic potential. Nevertheless, this does not exclude a role for epithelial–mesenchymal plasticity. Carcinoma cells frequently exhibit partial or hybrid phenotypic states wherein vimentin expression alone may not fully encapsulate the extent of EMT [14,15]. Future studies incorporating epithelial adhesion markers and EMT-associated transcription factors are necessary for comprehensive assessment.

To our knowledge, RUNX2 and osteocalcin expression has not been previously investigated in feline PPCs. Their evaluation was prompted by the metastasis to the distal phalanges characterizing feline lung-digit syndrome, now encompassed within feline MODAL syndrome, and the potential relevance of bone-associated cellular programs to metastatic colonization [6,7]. Although RUNX2 and osteocalcin are established regulators of normal bone biology, their pathological significance in feline pulmonary neoplasia was previously uncharacterized [18,19,20].

Osteocalcin expression was detected in the majority of PPCs but did not differ significantly between the metastatic and non-metastatic groups. Osteocalcin did not show a significant association with digital metastatic behavior in this group. It may represent a broader biological feature of these tumors, although a modest association cannot be definitively excluded due to the small sample size [29].

In contrast, RUNX2 expression was significantly elevated in PPCs associated with digital metastases. Beyond normal skeletal development, aberrant RUNX2 expression in human carcinomas is associated with invasive behavior, extracellular matrix remodeling, tumor progression, and metastasis [20,21,22,23,30,31,32,33,34,35]. While the present data support a significant association between increased RUNX2 expression and the presence of documented digital metastasis, this retrospective design does not establish a causal role for RUNX2 as a primary driver of metastatic dissemination. The critical distinction between a causal driver and a secondary marker remains unresolved in this context. It remains undetermined whether RUNX2 upregulation represents a secondary response to the bone microenvironment or plays another biological role. Further systematic comparative studies are required to clarify this relationship.

While the principal finding of this study is that primary pulmonary carcinomas in the metastatic group (PPC-DM) inherently exhibit significantly higher RUNX2 immunoreactivity than those in the non-metastatic group (PPC-NDM), the evaluation of individual paired trajectories revealed further notable changes during disease progression. Specifically, RUNX2 staining indices increased in every digital metastatic lesion compared to its corresponding primary tumor. Rather than reflecting a pre-existing phenotype that is merely maintained during dissemination, this consistent primary-to-metastasis upregulation strongly supports the hypothesis of clonal selection or further RUNX2 upregulation following metastatic colonization. Additionally, Ki-67 proliferation indices were consistently high in both groups and numerically greater in PPCs with digital metastases, potentially indicating increased proliferative activity in metastasizing tumors [12,13]. Several limitations of this study must be consolidated and acknowledged. First, the small sample size (*n* = 18), particularly the limited number of metastatic cases (*n* = 5), introduces a substantial risk of type II statistical errors and restricts our findings to exploratory associations. Second, the retrospective design and absence of standardized longitudinal staging protocols mean that occult metastases in the control group cannot be definitively excluded, potentially introducing misclassification bias. Third, although our initial study design focused exclusively on the lung-digit axis, the lack of available residual tissue for the documented extra-digital metastases (intestine, lymph node, subcutis) represents the main constraint of the existing material. Because these extra-digital lesions are precisely the comparator needed to distinguish general metastatic competence from digital or osseous specificity, the current study cannot confirm preferential digital colonization. This question remains unanswered, and further studies are required to distinguish general metastatic competence from specific tissue tropism. Furthermore, assessing vimentin expression alone provides only a partial characterization of EMT; comprehensive panels of EMT-associated transcription factors are required to draw definitive conclusions regarding cellular plasticity. Additionally, the lack of formal experimental antibody validation (e.g., Western blot) in feline FFPE tissues limits the definitive interpretation of our immunohistochemical signals. Finally, the descriptive, single-timepoint nature of this study lacks longitudinal survival data, precluding the direct linking of RUNX2 expression to patient prognosis or time to metastasis.

## 5. Conclusions

This study provides the first characterization of RUNX2 and osteocalcin expression in feline PPCs, including tumors associated with digital metastases. Increased RUNX2 expression was identified as a significant immunophenotypic feature of feline PPCs with digital metastases. In contrast, vimentin and osteocalcin expression did not differ between tumors with and without digital metastases, while Ki-67 indices were numerically higher in the digital metastasis group but did not reach statistical significance. In conclusion, this study demonstrates that significantly higher RUNX2 immunoreactivity is observed in primary pulmonary carcinomas from cats with documented digital metastasis compared to those without. Furthermore, paired evaluations revealed that RUNX2 expression increased even further in the digital metastatic lesions compared to their matched primary tumors. Due to the limitations regarding the unavailability of extra-digital metastatic tissues for comparison, these findings cannot establish preferential digital colonization or the metastatic behavior of the tumors. Ultimately, further molecular and functional studies are necessary to clarify the precise biological significance of RUNX2 and to determine whether it could serve as a prognostic indicator or therapeutic target in feline pulmonary oncology.

## Figures and Tables

**Table 1:** Primary antibodies and immunohistochemical conditions used for the evaluation of feline primary pulmonary carcinomas.

Antibody	Clone	Supplier	Dilution	Epitope Retrieval	Positive Control
MCK	AE1/AE3	LeicaBiosystems Newcastle Ltd., Newcastle, UK	RTU	Enzymatic	Feline lung (bronchiolar epithelium)
TTF-1	SPT 24	LeicaBiosystems Newcastle Ltd., Newcastle, UK	RTU	Citrate	Feline lung (type II pneumocytes and bronchioles)
Ki-67	MM1	LeicaBiosystems Newcastle Ltd., Newcastle, UK	RTU	Citrate	Feline lymph node (proliferating lymphoid cells)
Osteocalcin	OCG4	ThermoFischer Scientific Waltham, MA, US	1:500	Enzymatic	Feline bone (osteoblasts)
Vimentin	V9	LeicaBiosystems Newcastle Ltd., Newcastle, UK	RTU	Citrate	Feline lung (stromal cells)
RUNX2	F2	Sigma Aldirch Saint Louis, MO, US	1:200	EDTA	Feline bone (osteoblasts)

## Data Availability

The original contributions presented in this study are included in the article. Further inquiries can be directed at the corresponding author.

## Supplementary Materials

The following supporting information can be downloaded at: https://www.mdpi.com/article/10.3390/vetsci13090980/s1, Figure S1: Gross features of feline primary pulmonary tumors and digital metastases; Figure S2: Histological features of feline primary pulmonary carcinomas and digital metastases.

## Abbreviations

The following abbreviations are used in this manuscript:

ADC	Adenocarcinoma
ADS	Adenosquamous carcinoma
DAB	3,3′-diaminobenzidine
EDTA	Ethylenediaminetetraacetic acid
EMT	Epithelial–mesenchymal transition
FFPE	Formalin-fixed, paraffin-embedded
FLDS	Feline lung-digit syndrome
FN	Field number
H&E	Hematoxylin and eosin
HER2	Human epidermal growth factor receptor 2
HPF	High-power field
IQR	Interquartile range
K-RAS	Kirsten rat sarcoma viral oncogene homolog
MCK	Multi-cytokeratin
MODAL	Muscular, ocular, digital, aortic, lung
OC	Osteocalcin
PPC	Primary pulmonary carcinoma
PPC-DM	Primary pulmonary carcinoma with documented metastasis
PPC-NDM	Primary pulmonary carcinoma without documented metastasis
RUNX2	Runt-related transcription factor 2
SD	Standard deviation
SI	Staining index
TP53	Tumor protein 53
TTF-1	Thyroid transcription factor 1
VIM	Vimentin

## Appendix A

**Table A1:** Histological and Gross Tumor Characteristics.

Case Number	Histological Diagnosis	Intratumoral Necrosis	Vascular Invasion	Mitotic Index	Inflammation	Tumor Location
1T	ADS	Marked	Moderate	5/2.37 mm^2^	Marked	Unilateral (left caudal lobe)
2T	ADS	Marked	Few	7/2.37 mm^2^	Marked	Unilateral (left caudal lobe)
3T	ADC, papillary type	Moderate	Moderate	7/2.37 mm^2^	Mild	Unilateral (right cranial lobe)
4T	ADS	Moderate	Few	3/2.37 mm^2^	Marked	Unilateral (right caudal lobe)
5T	ADC, papillary type	Mild	Few	6/2.37 mm^2^	Mild	Unilateral (left caudal lobe)
6T	ADC, papillary type	Moderate	Few	5/2.37 mm^2^	Marked	Unilateral (left cranial lobe)
7T	ADC, papillary type	Moderate	Few	6/2.37 mm^2^	Moderate	Unilateral (right middle lobe)
8T	ADS	Marked	Few	3/2.37 mm^2^	Marked	Unilateral (left cranial lobe)
9T	ADS	Marked	Few	5/2.37 mm^2^	Mild	Unilateral (right cranial lobe)
10T	ADC, acinar type	Marked	Few	4/2.37 mm^2^	Marked	Unilateral (left caudal lobe)
11T	ADS	Marked	Few	3/2.37 mm^2^	Mild	Unilateral (left cranial lobe)
12T	ADC, acinar type	Moderate	Few	6/2.37 mm^2^	Mild	Unilateral (accessory lobe)
13T	ADS	Moderate	Few	4/2.37 mm^2^	Moderate	Unilateral (right caudal lobe)
1TM	ADS	Moderate	Few	4/2.37 mm^2^	Moderate	Unilateral (left caudal lobe)
2TM	ADC, papillary type	Marked	Few	6/2.37 mm^2^	Marked	Unilateral (left caudal lobe)
3TM	ADS	Marked	Few	6/2.37 mm^2^	Mild	Unilateral (right caudal lobe)
4TM	ADS	Moderate	Few	4/2.37 mm^2^	Mild	Unilateral (right middle lobe)
5TM	ADS	Marked	Few	6/2.37 mm^2^	Moderate	Unilateral (right cranial lobe)
1M	Metastasis (digit)—ADS	Moderate	Few	4/2.37 mm^2^	Marked	Thoracic limb
2M	Metastasis (digit)—ADC, papillary type	Moderate	Moderate	3/2.37 mm^2^	Mild	Pelvic limb
3M	Metastasis (digit)—ADS	Moderate	Moderate	6/2.37 mm^2^	Mild	Pelvic limb
4M	Metastasis (digit)—ADS	Moderate	Moderate	5/2.37 mm^2^	Mild	Pelvic limb
5M	Metastasis (digit)—ADS	Moderate	Moderate	8/2.37 mm^2^	Mild	Pelvic limb

Abbreviations and Study Groups: T (Cases 1T–13T): Primary pulmonary carcinoma without metastasis (n=13). TM (Cases 1TM–5TM): Primary pulmonary carcinoma with confirmed digital metastasis (n=5). M (Cases 1M–5M): Matched metastatic lesion (digit) corresponding to the respective primary tumor in the TM group (n=5). ADC: Adenocarcinoma. ADS: Adenosquamous carcinoma.

**Table A2:** Immunohistochemical Profiling (MCK, TTF-1, Vim, OC, RUNX2, and Ki-67).

Case Number	MCK	TTF-1	Vimentin (Score)	Osteocalcin (Score)	RUNX2 (SI)	Ki-67 (%)
1T	+	+	1	3	2	72.54%
2T	+	+	4	3	2	69.14%
3T	+	+	3	3	2	62.90%
4T	+	+	3	3	2	56.20%
5T	+	+	3	1	6	76.11%
6T	+	+	1	3	4	74.12%
7T	+	+	1	2	2	61.05%
8T	+	+	2	1	2	19.60%
9T	+	+	3	0	4	88.63%
10T	+	+	2	2	2	61.96%
11T	+	+	1	3	4	72.12%
12T	+	+	1	2	8	66.60%
13T	+	+	3	3	4	71.69%
1TM	+	+	4	3	8	78.60%
2TM	+	+	2	2	9	76.11%
3TM	+	+	2	3	4	80.03%
4TM	+	+	3	3	8	65.49%
5TM	+	+	1	0	8	78.69%
1M	+	+	4	3	12	59.45%
2M	+	+	2	3	12	78.19%
3M	+	+	4	3	9	52.64%
4M	+	+	3	3	9	62.96%
5M	+	+	3	3	9	51.87%

Abbreviations and Study Groups: T (Cases 1T–13T): Primary pulmonary carcinoma without metastasis (n=13). TM (Cases 1TM–5TM): Primary pulmonary carcinoma with confirmed digital metastasis (n=5). M (Cases 1M–5M): Matched metastatic lesion (digit) corresponding to the respective primary tumor in the TM group (n=5). Note: Numeric prefixes designate individual animals within each group (e.g., case 1M is the matched metastasis for primary tumor 1TM).

## References

[1] D’Costa S., Yoon B.-I., Kim D.-Y., Motsinger-Reif A.A., Williams M., Kim Y. (2012). Morphologic and molecular analysis of 39 spontaneous feline pulmonary carcinomas. Vet. Pathol..

[2] Ludwig L., Dobromylskyj M., Wood G.A., van der Weyden L. (2022). Feline oncogenomics: What do we know about the genetics of cancer in domestic cats?. Vet. Sci..

[3] Santos I.R., Raiter J., Lamego É.C., Bandinelli M.B., Dal Pont T.P., Siqueira K.F., Almeida B.A., Panzeira W., Sonne L., Driemeier D. (2023). Feline pulmonary carcinoma: Gross, histological, metastatic, and immunohistochemical aspects. Vet. Pathol..

[4] Wilson D.W., Meuten D.J. (2016). Tumors of the respiratory tract. Tumors in Domestic Animals.

[5] Hahn K.A., McEntee M.F. (1998). Prognosis factors for survival in cats after removal of a primary lung tumor: 21 cases (1979–1994). Vet. Surg..

[6] Goldfinch N., Argyle D.J. (2012). Feline lung–digit syndrome: Unusual metastatic patterns of primary lung tumours in cats. J. Feline Med. Surg..

[7] Thrift E., Greenwell C., Turner A.-L., Harvey A.M., Maher D., Malik R. (2017). Metastatic pulmonary carcinomas in cats (‘feline lung–digit syndrome’): Further variations on a theme. J. Feline Med. Surg. Open Rep..

[8] Grossman D.A., McNiel E.A., Hackett T.B., Barsky S.H. (2002). Establishment of an immortalized cell line and transplantable xenograft from a bronchioloalveolar lung carcinoma of a cat. Am. J. Vet. Res..

[9] Muscatello L.V., Di Oto E., Dignazzi M., Murphy W.J., Porcellato I., De Maria R., Raudsepp T., Foschini M.P., Sforna M., Benazzi C. (2021). HER2 Overexpression and Amplification in Feline Pulmonary Carcinoma. Vet. Pathol..

[10] Ramos-Vara J.A., Miller M.A., Johnson G.C. (2005). Usefulness of Thyroid Transcription Factor-1 Immunohistochemical Staining in the Differential Diagnosis of Primary Pulmonary Tumors of Dogs. Vet. Pathol..

[11] Kujawa A., Olias P., Böttcher A., Klopfleisch R. (2014). Thyroid transcription factor-1 is a specific marker of benign but not malignant feline lung tumours. J. Comp. Pathol..

[12] Ishibashi N., Maebayashi T., Aizawa T., Sakaguchi M., Nishimaki H., Masuda S. (2017). Correlation between the Ki-67 proliferation index and response to radiation therapy in small cell lung cancer. Radiat. Oncol..

[13] Sabattini S., Lopparelli R.M., Rigillo A., Giantin M., Renzi A., Matteo C., Capitani O., Dacasto M., Mengoli M., Bettini G. (2018). Canine Splenic Nodular Lymphoid Lesions: Immunophenotyping, Proliferative Activity, and Clonality Assessment. Vet. Pathol..

[14] Thiery J.P., Acloque H., Huang R.Y., Nieto M.A. (2009). Epithelial-mesenchymal transitions in development and disease. Cell.

[15] Zeisberg M., Neilson E.G. (2009). Biomarkers for epithelial-mesenchymal transitions. J. Clin. Investig..

[16] Dauphin M., Barbe C., Lemaire S., Nawrocki-Raby B., Lagonotte E., Delepine G., Birembaut P., Gilles C., Polette M. (2013). Vimentin expression predicts the occurrence of metastases in non-small cell lung carcinomas. Lung Cancer.

[17] Agustina H., Asyifa I., Hernowo B.S. (2018). The Role of Osteocalcin and Alkaline Phosphatase Immunohistochemistry in Osteosarcoma Diagnosis. Pathol. Res. Int..

[18] Martiniakova M., Mondockova V., Biro R., Kovacova V., Babikova M., Zemanova N., Ciernikova S., Omelka R. (2023). The link between bone-derived factors osteocalcin, fibroblast growth factor 23, sclerostin, lipocalin 2 and tumor bone metastasis. Front. Endocrinol..

[19] Garg S., Chaurasia J.K., Kaushal D., Jayashankar E., Yadav S.K., Joshi D., Walke V. (2026). High Immunohistochemical Expression of Runt-Related Transcription Factor 2 (RUNX2) Is Associated With High Tumor Grade, Muscle Invasion, Lymph Node Metastasis, and Advanced Stage in Urinary Bladder Cancer. Cureus.

[20] Si W., Kan C., Zhang L., Li F. (2023). Role of RUNX2 in breast cancer development and drug resistance. Oncol. Lett..

[21] Herreño A.M., Ramírez A.C., Chaparro V.P., Fernandez M.J., Cañas A., Morantes C.F., Moreno O.M., Brugés R.E., Mejía J.A., Bustos F.J. (2019). Role of RUNX2 transcription factor in epithelial mesenchymal transition in non-small cell lung cancer lung cancer: Epigenetic control of the RUNX2 P1 promoter. Tumour Biol..

[22] Li H., Zhou R.J., Zhang G.Q., Xu J.P. (2013). Clinical significance of RUNX2 expression in patients with nonsmall cell lung cancer: A 5-year follow-up study. Tumour Biol..

[23] Bernal C., Otalora A., Cañas A., Barreto A., Prieto K., Montecino M., Rojas A. (2022). Regulatory Role of the RUNX2 Transcription Factor in Lung Cancer Apoptosis. Int. J. Cell Biol..

[24] The UniProt Consortium (2023). UniProt: The universal protein knowledgebase in 2023. Nucleic Acids Res..

[25] Altschul S.F., Gish W., Miller W., Myers E.W., Lipman D.J. (1990). Basic local alignment search tool. J. Mol. Biol..

[26] Sievers F., Wilm A., Dineen D., Gibson T.J., Karplus K., Li W., Lopez R., McWilliam H., Remmert M., Söding J. (2011). Fast, scalable generation of high-quality protein multiple sequence alignments using Clustal Omega. Mol. Syst. Biol..

[27] Happ M., Bathke A.C., Brunner E. (2018). Optimal sample size planning for the Wilcoxon-Mann-Whitney test. Stat. Med..

[28] Divine G., Norton H.J., Hunt R., Dienemann J. (2013). A review of analysis and sample size calculation considerations for Wilcoxon tests. Anesth. Analg..

[29] Kim G.Y., Kim J., Kim T.S., Han J. (2009). Pulmonary adenocarcinoma with heterotopic ossification. J. Korean Med. Sci..

[30] Huang B., Liu H., Chan S., Liu J., Gu J., Chen M., Kuang L., Li X., Zhang X., Li J. (2023). RUNX2 promotes the suppression of osteoblast function and enhancement of osteoclast activity by multiple myeloma cells. Med. Oncol..

[31] Zhao W., Yang H., Chai J., Xing L. (2021). RUNX2 as a promising therapeutic target for malignant tumors. Cancer Manag. Res..

[32] Xiao D., Liu K., Chen J., Gong Y., Zhou X., Huang J. (2021). RUNX2 as a Potential Prognosis Biomarker and New Target for Human Lung Cancer. Explor. Res. Hypothesis Med..

[33] Wu S., Pan Y., Mao Y., Chen Y., He Y. (2021). Current progress and mechanisms of bone metastasis in lung cancer: A narrative review. Transl. Lung Cancer Res..

[34] Lin T.-C. (2023). RUNX2 and cancer. Int. J. Mol. Sci..

[35] Zhang F., Cho W.C. (2023). Therapeutic potential of RUNX1 and RUNX2 in bone metastasis of breast cancer. Expert Opin. Ther. Targets.

