# Supplementary material for: Immunohistochemical Evaluation of RUNX2, Osteocalcin, and Vimentin in Feline Primary Pulmonary Carcinomas
Source: Vet Sci. 2026 Sep 17;13(9):980. doi: 10.3390/vetsci13090980 (PMC13611842; doi:10.3390/vetsci13090980)
Supplement: Supplementary file 1 [file vetsci-13-00980-s001.zip › vetsci-4527771-supplementary.pdf]

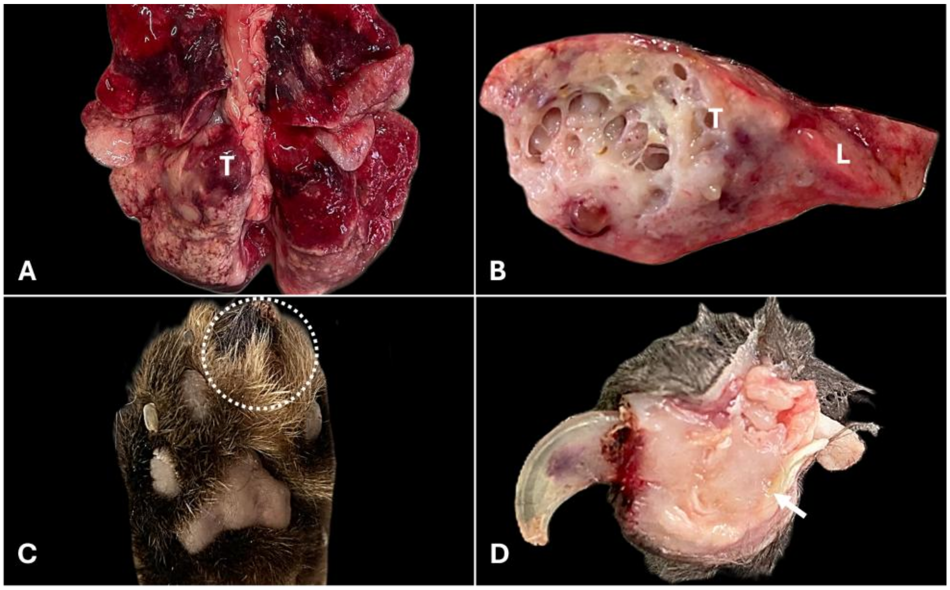

Figure S1. Gross features of feline primary pulmonary tumors and digital metastases. (**A**) Tan to red, poorly demarcated, infiltrative neoplastic mass within the left caudal lung lobe. (**B**) Cut surface of a pulmonary tumor showing extensive necrotic areas with cavitation. (**C**) Digital metastatic lesion involving the distal phalanx, characterized by marked enlargement of the digit no. 3, and nail-bed ulceration with crust formation (dashed circle). (**D**) Sagittal section of an affected digit showing an unencapsulated, white to tan metastatic mass (arrow) extensively infiltrating the surrounding soft tissues and replacing the osseous architecture of the distal phalanx. Abbreviations: T, tumor; L, non-neoplastic lung parenchyma

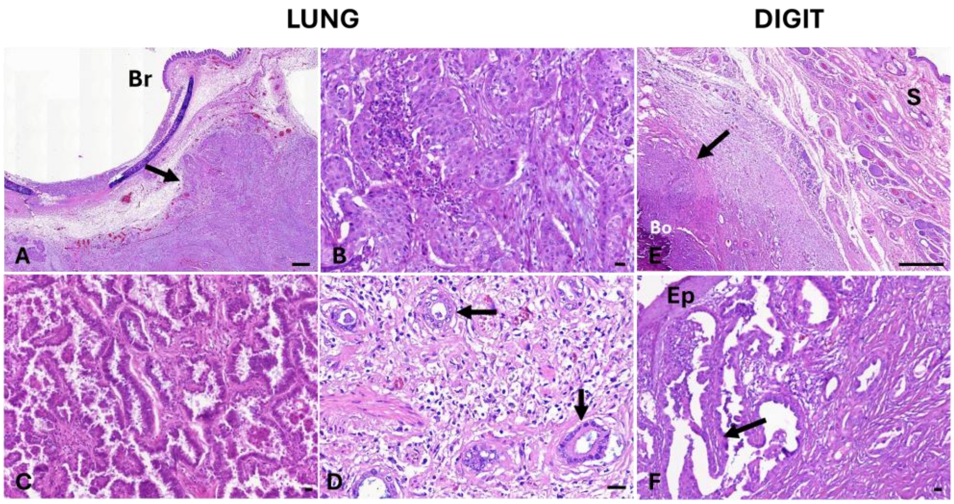

Figure S2. Histological features of feline primary pulmonary carcinomas and digital metastases. (A) Densely cellular, unencapsulated neoplastic mass (arrow) infiltrating the pulmonary parenchyma adjacent to a bronchus, scale bar=20μm. **(B)** Adenosquamous carcinoma exhibiting biphasic differentiation, with a malignant glandular component and polygonal neoplastic cells showing squamous differentiation, scale bar=20μm. **(C)** Papillary adenocarcinoma showing papillary projections lined by cuboidal to columnar neoplastic cells and supported by delicate fibrovascular cores, scale bar=20μm. **(D)** Acinar adenocarcinoma characterized by neoplastic epithelial cells arranged in distinct acinar structures with central lumina, scale bar=20μm. **(E)** Digital metastatic tumor consisting of unencapsulated neoplastic tissue (arrow) infiltrating the subepidermal connective tissue and partially replacing the phalangeal bone, scale bar=20μm. **(F)** Digital metastasis showing invasive neoplastic epithelial cells (arrow) forming irregular papillary structures within the dermis, scale bar=20μm. Abbreviations: Br-bronchus, Bo-bone, Ep-epidermis, S-skin; Scale bar = 20μm
